# Giant inguinal melanoma treated with complex oncological and reconstructive surgery: A case report

**DOI:** 10.1016/j.amsu.2022.104320

**Published:** 2022-08-02

**Authors:** Marco Rastrelli, Claudia Di Prata, Paolo Del Fiore, Marta Sbaraglia, Vincenzo Vindigni, Franco Bassetto

**Affiliations:** a Soft-Tissue, Peritoneum and Melanoma Surgical Oncology Unit, Veneto Institute of Oncology IOV-IRCCS, Padua, Italy; b Department of Surgery Oncology and Gastroenterology (DISCOG), University of Padova, Padova, Italy; c Pathological Anatomy Unit, University Hospital of Padua, Padua, Italy; d Clinic of Plastic Surgery, Department of Neuroscience, Padua University Hospital, University of Padua, Padua, Italy

**Keywords:** Melanoma, Giant melanoma, Multidisciplinary approach, Reconstructive surgery, Case report

## Abstract

A 53-year-old woman came to our attention with a giant inguinal mass that was growing in the last 5 months, so she underwent a total body CT-scan that showed a 25 × 16 × 21cm mass of the right inguinal region which was compressing the femoral vessels and infiltrated the omolateral rectum muscle, pulmonary embolism and thrombosis of the right femoral vein. We performed a tru-cut biopsy that was consistent with an undifferentiated round-cell sarcoma. So, we performed a wide excision of the mass and a reconstruction with a pedunculated muscular flap of the tensor muscle of the fasciae late, a graft of omologous fasciae late and a graft of the great saphena vein onto the superficial femoral artery. The histological examination of the specimen demonstrated instead an “*atypical amelanotic sarcomatoid malignant melanoma*” with rabdoid aspects. The patient underwent a radicalization surgery and reconstruction with microsurgical great dorsal and anterior serratus flap. To our knowledge, this is the biggest inguinal melanoma treated with surgical excision described so far.

## Highlights

• Giant primary melanoma is generally accepted as greater than 10 cm, and almost associated with extensive metastatic disease.
• It is important the patients don't present with such a clinical challenge, the surgery could impact the quality of life.
• Patients with growing mass must be referred quickly to a diagnosis to obtain a less extent of surgical intervention.

## 1 Introduction and importance

The giant primary melanoma is generally accepted as “greater than 10cm”, and is almost exclusively associated with extensive metastatic disease. It is important to reduce the number of patients that present themselves with such a clinical challenge, because the extent of the surgical demolition could impact the quality of life, since in some cases it could lead to even limb or organ amputation. In our case, the mass was spreading to the exterior genital organs and we had to perform a wide excision over the vulvar labia majora. This case report has been reported in line with the SCARE criteria [1].

## 2 Case presentation

**Fig. 1:**
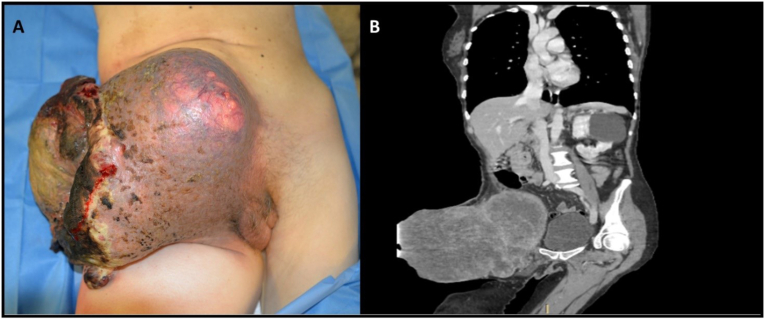
A: Pre-treatment Giant Melanoma; B: Pre-treatment CT imaging Giant Melanoma.

A 53 years-old female, after an accidental fall, was visited at the emergency department, where it was discovered a mass that the patient described as a neoformation that had been growing in the previous 5 months (Fig. 1A). She underwent a total body contrast-enhanced computed tomography (CT) that showed a 25 × 16 × 21 cm mass of the right inguinal region which was compressing the femoral vessels and infiltrated the homolateral rectus abdominis muscle. Other findings were a pulmonary embolism and a thrombosis of the right femoral vein (Fig. 1B). The patient was then referred to the Melanoma and Sarcoma unit of Veneto Institute of Oncology. At physical examination, a giant, hard, painful, and fixed inside the muscles, mass was found. We therefore performed a tru-cut biopsy of the neoformation that was consistent with a diagnosis of undifferentiated round-cell sarcoma [2,3]. We discussed the case with our sarcoma multidisciplinary team, and we proposed a surgical excision since the disease was locally advanced, but not spread to other sites. We performed a wide excision of the mass and a reconstruction with a pedunculated muscular flap of the tensor fasciae latae muscle, a graft of homologous fascia late and a graft of the great saphena vein onto the superficial femoral artery, that was sacrificed during the excision due to tumour infiltration. We did not directly perform a skin closure of the defect, but we applied a dermal substitute while the surgical specimen was analysed.

**Fig. 2:**
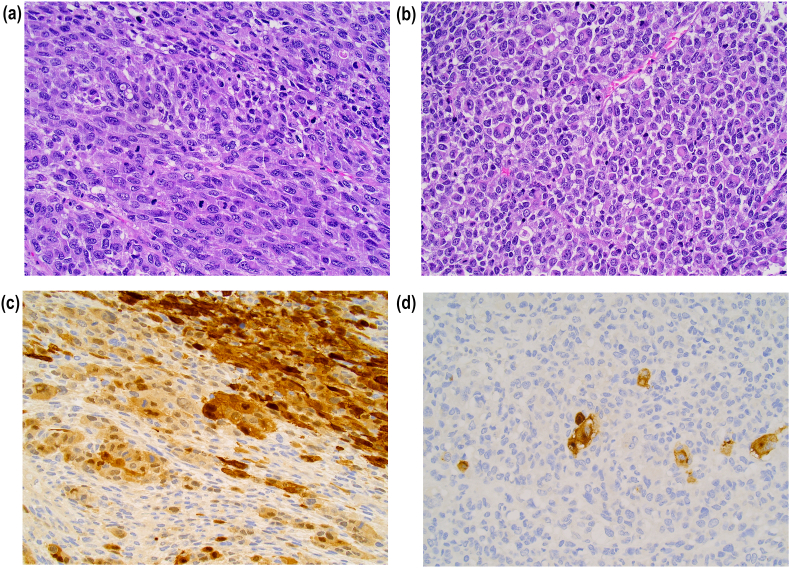
(A–D): Highly aggressive tumor composed of spindle and pleomorphic neoplastic cells (A) mixed with areas showing rhabdoid features (B). Multifocal immunopositivity for S100 (C) associated with staining for HMB45 in isolated neoplastic cells (D) support the diagnosis of sarcomatoid malignant melanoma.

The histological examination of the specimen demonstrated not a sarcoma, but a rare case of an atypical amelanotic sarcomatoid malignant melanoma with rabdoid aspects (Fig. 2A–D), with a close medial (vulvar) margin [[4], [5], [6], [7], [8]]. The final diagnosis was changed, and we completed the exams with an ultrasound scan of inguinal and axillary regions that resulted negative. A full skin examination and dermoscopy were performed and both resulted also negative. Serum protein S-100 B was normal; serum lactate dehydrogenase was normal. The specimen was then tested for BRAF mutation. The BRAF sequencing not indicated a V600 mutation.

**Fig. 3:**
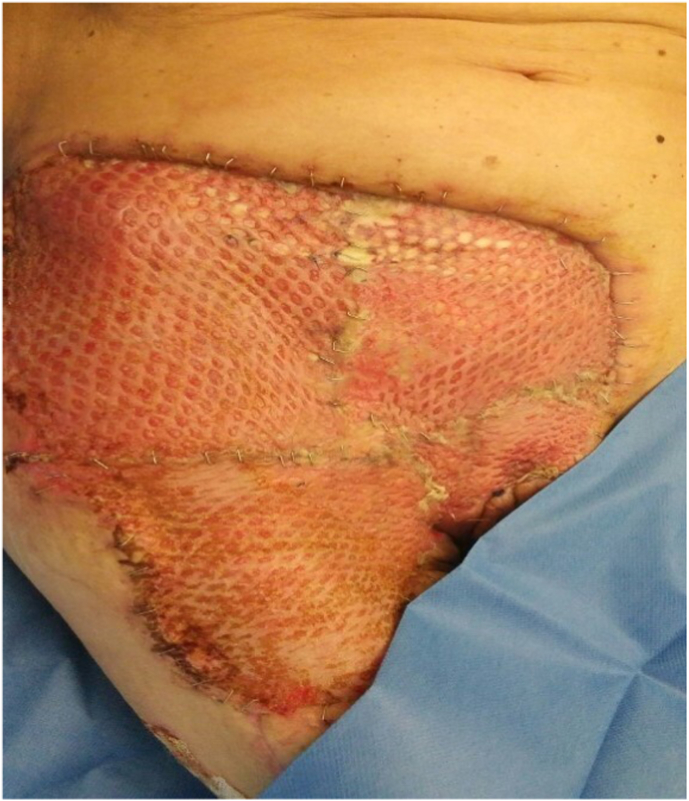
Post radicalization surgery and a surgical reconstruction with microsurgical great dorsal and anterior serratus flap and a homologous skin graft.

At this moment we planned to perform the radicalization surgery and subsequently the closing of the defects in the same operating session. Therefore, the patient underwent a radicalization surgery and a surgical reconstruction (Fig. 3) with a microsurgical great dorsal and anterior serratus flap and a homologous skin graft [9,10].

## 3 Clinical discussion

To our knowledge, this is the biggest melanoma treated with surgical excision described so far. The adjective “giant” referred to melanoma is generally accepted as “greater than 10cm”, and in our case the mass was a very large one. It is important to reduce the number of patients that present themselves with such a clinical challenge, because the extent of the surgical demolition could impact the quality of life, since in some cases it could lead to even limb or organ amputation. In our case, the mass was spreading to the exterior genital organs and we had to perform a wide excision over the vulvar labia majora.

The largeness of the mass impairs not only the demolition, but also the reconstruction of the defect. Using a dermal substitute, we could obtain two different advantages: on one hand, in a more reconstructive setting, we could partly reduce the greatness of the defect, since it has been demonstrated that dermal substitute has an impact on the process of scar tissue forming. On the other hand, in a more oncological aspect, we could delay the defect closure while waiting for the histological analysis of the specimen, since it is important to spare skin that can be used for a definitive closure after a radicalization, if it is already predicted that a wide excision has to be performed.

## 4 Conclusion

In conclusion, patients with growing mass must be referred to a medical examination to arrive at a diagnosis while the mass itself is more manageable in terms of both demolition and reconstruction to obtain a less extent of surgical intervention, not only in an oncological setting, but also to maintain a higher quality of life. Since these interventions have a higher risk of complication and pitfalls, it is mandatory to discuss these unique cases in a multidisciplinary approach.

## Ethical approval

This case report is exempt from ethical approval.

## Sources of funding

This research received funds to “Ricerca Corrente 2022” to cover publication costs.

## Author contribution

MR, CDP wrote the report; PDF prepared the figures and re-viewed the report. PDF provided the pathology images and interpretation. VV, FB and MS supervised as the attending physicians in the care of this patient, and approved the final version of the report.

## Registration of research studies

Not applicable.

## Guarantor

Dr.Marco Rastrelli.

## Consent

Written informed consent was obtained from the patient for publication of this report and any accompanying images. A copy of the written consent is available for review by the Editor-in-Chief of this journal on request.

## Provenance and peer review

Not commissioned, externally peer-reviewed.

## Declaration of competing interest

The authors declared no potential conflicts of interest with respect to the research, authorship, and/or publication of this article.
